# Implicit Learning of Stimulus Regularities Increases Cognitive Control

**DOI:** 10.1371/journal.pone.0093874

**Published:** 2014-04-15

**Authors:** Jiaying Zhao, Devin Karbowicz, Daniel Osherson

**Affiliations:** 1 Department of Psychology and Institute for Resources, Environment and Sustainability, University of British Columbia, Vancouver, BC, Canada; 2 Department of Psychology, Cornell University, Ithaca, New York, United States of America; 3 Department of Psychology, Princeton University, Princeton, New Jersey, United States of America; University of California, San Francisco, United States of America

## Abstract

In this study we aim to examine how the implicit learning of statistical regularities of successive stimuli affects the ability to exert cognitive control. In three experiments, sequences of flanker stimuli were segregated into pairs, with the second stimulus contingent on the first. Response times were reliably faster for the second stimulus if its congruence tended to match the congruence of the preceding stimulus, even though most participants were not explicitly aware of the statistical regularities (Experiment 1). In contrast, performance was not enhanced if the congruence of the second stimuli tended to mismatch the congruence of the first stimulus (Experiment 2). The lack of improvement appears to result from a failure of learning mismatch contingencies (Experiment 3). The results suggest that implicit learning of inter-stimulus relationships can facilitate cognitive control.

## Introduction

The Eriksen flanker task requires the identification of a central target in the presence of surrounding distractors [1]. Arrowheads are typically used, yielding stimuli like these:


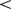
 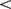 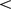 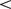 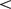 (correct answer: “left”).


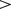
 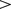 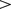 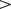 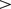 (correct answer: “right”).


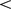
 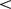 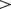 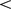 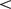 (correct answer: “right”).


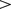
 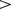 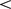 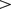 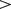 (correct answer: “left”).

The first two stimuli in are termed *congruent*, the last two *incongruent*. We will call two successive stimuli in the flanker task *concordant* if they are matched for congruence, that is, either each is drawn from the top two rows of (1), or each is drawn from the bottom two rows. Two successive stimuli are *discordant* if they are not concordant, that is, one is drawn from the top two rows of (1) and one from the bottom. Thus, congruence and incongruence are properties of individual stimuli whereas concordance and discordance are properties of pairs. Note that the two members of a concordant pair may or may not require the same answer, and likewise for discordance.

It is well documented that response times (RTs) are lower for congruent compared to incongruent stimuli [1], [2]. It has also been found that RTs are lower for the second stimulus of concordant pairs compared to the second stimulus of discordant pairs (the *Gratton effect*, [2]). One possible mechanism for the latter phenomenon is that congruent stimuli increase attention to surrounding flankers in the subsequent stimulus, thereby offering a more extended visual target in case of congruence but increasing interference in case of incongruence. Likewise, incongruent stimuli would draw attention away from flankers, thereby slowing the response to a following congruent stimulus but limiting interference in case of incongruence. Concordance would thus enhance performance in both situations, compared to discordance. In another version of the experiment [2], [3], an explicit cue signaled the congruence/incongruence of subsequent stimuli. RTs were lower when cues predicted congruent stimuli, but no difference in RT was observed for cues predicting incongruent stimuli.

Recent evidence suggests that the Gratton effect hinges on concordant pairs with the same correct answer, that is, on successive stimuli that are *identical*. RT appears not to decrease for the second member of a concordant pair that requires a different answer than the first [4]–[6]. The Gratton effect may thus reflect mere repetition priming rather than priming for the more abstract property of stimulus congruence or incongruence.

Perhaps a more robust Gratton effect can be achieved through learning implicitly the statistical structure of successive flanker stimuli, instead of relying on explicit cuing. This speculation is motivated by findings on preparatory control in task switching. In a predictable alternating-runs paradigm, participants are able to learn to prepare for the upcoming stimulus and reduce switch cost (see, for example, [7], and [8] for a review). In the first two experiments reported below, statistical regularities are implicitly embedded in the stimuli. Specifically, the congruency of the second member of a pair is contingent on that of the first, unbeknownst to the participants. Importantly, the response required by either stimulus in the pair was randomly determined, which ensures that any observed effect is not due to repetition priming, but rather driven by learning of the abstract property of stimulus concordance or discordance.

The goal of the current study is to examine how the implicit learning of regularities between successive stimuli influence the control of attention. In all experiments, two flanker stimuli are presented in a pair for every trial. Unbeknownst to the participants, the congruency of the second stimulus is predictable from that of the first. In Experiment 1, the congruency of the second stimulus tends to remain the same as for the first (e.g., a congruent trial tends to follow another congruent trial). In Experiment 2, the congruency of the second stimulus tends to differ (e.g., a congruent trial tends to follow an incongruent trial). In all experiments, the overall percentage of congruent and incongruent trials is roughly the same (i.e., 50%). We hypothesize that the implicit learning of congruency between two stimuli will lead to faster response in the second stimulus in a pair.

## Experiment 1

### Methods

#### Participants

Sixty adults from Mercer County, New Jersey, were tested individually in return for $5 compensation (39 female, mean age 22.5 yrs, SD = 2.8). All experiments reported here have been approved by the Princeton University Institutional Review Board. Written consent was obtained from every participant.

#### Materials and Procedure

**Figure 1:**
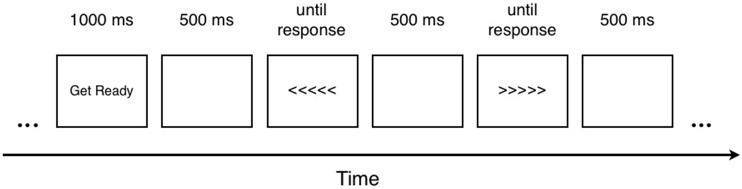
Sample trial in Experiment 1.

Each trial consisted of a pair of stimuli presented sequentially. Stimuli were as shown in (1), presented at fixation on a computer monitor, occupying approximately two visual degrees. The trial began with the sign “Get Ready” displayed at the center of the screen for 1 second, followed by a blank screen for 500 ms. The first stimulus in a pair was then presented at the center of the screen until the participant responded. After response, a blank screen appeared for 500 ms. followed by the second stimulus which was presented until the participant responded again. Finally, a blank screen appeared for 500 ms. before the onset of the next trial. An example trial is shown in Figure 1.

There were two conditions in the experiment, called *concordant* versus *random*; they were performed by separate groups of thirty participants each (uninformed of the condition they were in). Each condition was composed of 200 trials, where each trial consisted of a pair of stimuli, as described above. In the concordant condition, 80% of the trials consisted of concordant pairs, 20% discordant. Half of the concordant trials involved congruent pairs, and the other half incongruent. Thus, if the first stimulus in a pair was congruent, there was an 80% probability that the second was also congruent. Likewise, if the first stimulus was incongruent, there was an 80% probability that the second was also incongruent. Within these constraints, all stimuli were chosen randomly. The first two columns of Table 1 lists all concordant pairs. Crucially, although the congruency of the pairs was manipulated, the central arrow direction was always randomly determined for every stimulus. This implies that participants could learn to predict the congruency of the second stimulus of a pair based on the first, but they could not learn to predict the specific arrow direction of the second stimulus. In the random condition, the concordance of every pair was randomly determined. In other words, the congruency of the first stimulus in a pair was not predictive of the congruency of the second stimulus. Just as for the concordant condition, in the random condition the central arrow direction was randomly determined for every stimulus in every pair.

**Table 1:** Average accuracy (%) and RT (ms.) for the second stimulus in concordant pairs from Experiment 1.

Pair		Concordant		Random		Significance
1st stim.	2nd stim.	Accu.	RT	Accu.	RT	RT
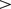 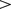 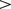 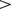 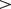	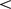 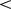 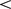 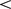 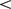	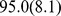	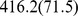	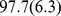	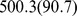	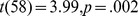
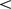 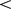 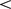 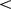 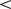	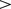 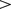 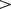 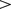 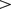	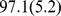	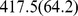	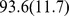	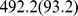	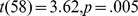
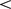 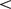 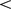 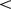 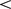	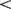 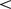 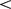 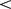 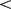	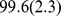	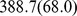	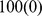	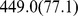	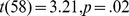
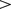 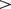 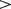 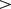 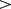	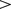 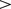 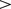 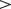 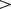	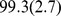	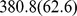	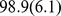	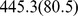	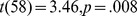
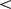 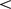 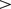 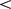 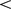	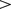 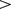 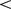 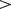 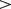	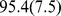	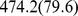	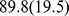	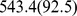	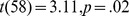
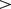 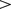 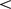 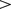 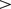	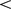 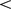 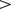 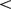 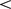	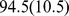	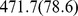	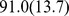	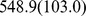	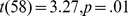
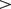 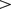 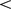 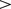 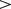	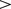 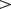 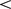 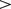 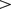	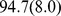	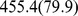	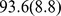	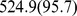	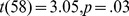
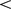 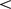 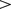 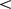 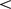	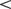 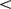 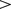 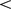 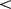	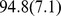	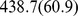	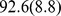	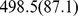	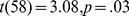

(Standard deviations are in parentheses; 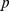 values are corrected for multiple-comparisons using the Bonferroni method).

Participants performed five practice trials before starting the experiment. They were instructed to indicate the direction of the middle arrow by pressing the “1” key or the “0” key for left and right, respectively. Participants were required to respond as accurately and quickly as possible.

### Results and Discussion

If participants in the concordant condition learned to exploit its congruency structure then these participants would be better prepared for the second stimulus in a pair, compared to participants in the random condition. Since learning might require several trials, however, we examined performance only on trials 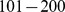 in each condition. Moreover, only a subset of these latter trials were included in the analysis. Specifically, in the concordant condition, we included only the 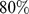 of trials that exhibited the same congruency for the two stimuli (both congruent or both incongruent). Likewise, in the random condition, only the concordant trials were selected (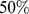 of trials). It was then possible to compare accuracy and RT between the two conditions with respect to the very same stimuli. For example, performance on the second stimulus of the concordant trial 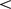 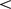 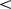 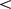 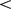 followed by 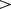 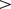 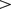 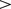 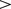 was compared to performance on the second stimulus of the identical trial in the random condition. (Performance on the first stimulus in a pair was ignored.) Finally, for every trial, accuracy was calculated by comparing whether the response matched the central arrow. For every participant and separately for congruent and incongruent trials, RTs more than 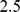 standard deviations above the mean were excluded from the analysis. Only 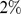 of the trials were removed as a result.

Average accuracy and RT for the second stimulus of concordant pairs in both the concordant and random conditions are presented in Table 1. Accuracy and RT were analyzed with a two-way mixed-design ANOVA (between-subjects factor: concordant vs. random condition; within-subjects factor: congruent vs. incongruent trials). For RT, there was a main effect of condition [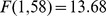, 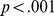], and also of congruency [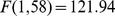, 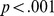]. For accuracy, there was a main effect of congruency [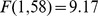, 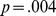], but not of condition [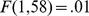, 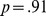]. There was no reliable interaction for accuracy or RT [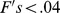, 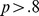]. We also conducted a pair-wise comparison. For each row in Table 1, concordant and random conditions were compared via independent-sample 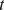-tests (corrected for multiple comparisons). For every one of the 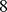 types of concordant trials, RT was reliably and consistently lower in the concordant condition than in the random condition. Despite the improvements in RT, only six of the 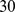 participants in the concordant condition were aware of the statistical relationship between the first and the second stimuli. The same pattern of results was observed when the six participants were excluded from the analysis. To further support the role of learning, we analyzed RT of the first 100 trials. Since the implicit learning process occurs over time, the effect in the first half of the experiment may not be as strong as that in the second half. Indeed, we found that the RT difference was smaller between the concordant and the random conditions [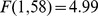, 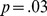] in the first 100 trials. There was a reliable interaction between trials (first vs. second 100 trials) and condition [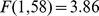, 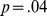]. These results suggest that participants learned to exploit the partial predictability of congruence in the concordant condition, focussing attention adaptively for the second stimulus in a trial. The substantially lower RTs seen in Table 1 for congruent compared to incongruent trials may reflect the advantage accruing to spreading attention across multiple arrowheads with the same message.

## Experiment 2

Experiment 1 documents the ability to exploit concordance in sequential stimuli but leaves open the same question about discordance. In a discordant pair if the first stimulus is congruent then the second is incongruent, and vice versa. Experiment 2 was isomorphic to the first except that it involved a discordant condition in place of the original concordant condition. The discordant condition was composed of 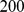 trials, 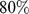 of which were discordant pairs, 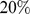 concordant. Half of the discordant trials involved a congruent stimulus followed by incongruent, and the reverse for the other half. As before, the central arrow direction was always randomly determined for every stimulus. For the random condition, the data from Experiment 1 were used again. Thirty new participants were recruited for Experiment 2, drawn from the same pool as before (21 female, mean age 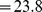 yrs, SD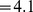).

### Results and Discussion

We compared performance on matching second stimuli in the discordant versus random conditions, taking into account just trials 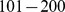. As before, only a subset of the trials were included in the analysis. Specifically, in the discordant condition, we included only the 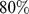 of trials that exhibited different congruency for the two stimuli. Likewise, in the random condition, only the discordant trials were selected (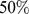 of trials). Trials with RTs beyond 2.5 standard deviations of the mean for every participant and for every congruency level were excluded. Only 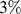 of the trials were removed as a result.

Accuracy and RT for the second stimulus of discordant pairs in the two conditions are presented in Table 2. As in Experiment 1, accuracy and RT were analyzed using a two-way mixed-design ANOVA (between-subjects factor: discordant vs. random condition; within-subjects factor: congruent vs. incongruent trials). For RT, there was a main effect of congruency [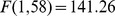, 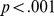], but not of condition [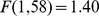, 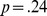]. For accuracy, there was a main effect of congruency [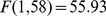, 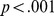], but not of condition [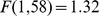, 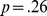]. There was no reliable interaction for accuracy or RT [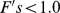, 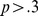]. Pair-wise comparisons revealed that for none of the eight types of discordant trials was RT reliably lower in the discordant compared to random condition; there were also no reliable differences in accuracy. Notice, however, that for all types of trials, the RTs were numerically lower in the discordant compared to random condition. Only four of the 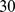 participants in the discordant condition noticed the statistical relationship, and the results remained the same without the four participants. The results show that there was no RT benefit in the second stimulus for discordant pairs.

**Table 2:** Average accuracy (%) and RT (ms.) for the second stimulus in discordant pairs from Experiment 2.

Pair		Discordant		Random	
1st stim.	2nd stim.	Accu.	RT	Accu.	RT
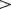 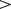 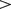 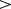 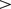	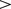 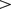 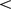 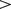 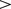	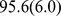	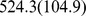	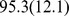	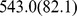
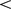 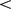 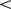 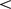 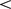	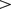 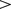 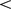 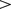 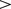	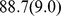	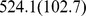	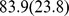	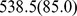
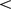 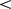 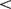 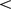 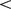	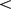 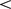 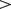 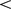 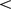	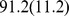	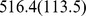	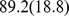	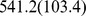
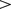 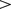 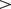 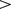 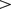	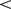 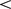 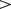 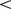 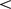	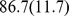	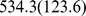	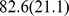	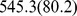
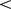 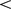 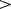 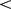 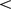	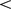 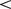 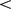 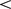 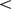	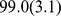	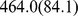	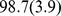	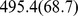
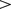 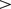 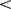 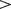 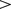	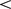 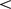 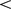 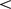 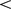	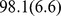	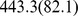	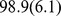	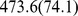
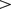 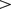 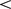 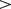 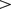	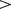 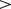 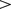 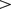 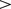	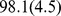	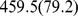	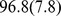	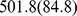
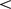 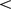 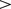 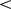 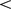	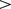 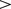 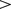 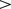 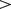	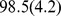	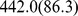	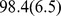	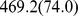

(Standard deviations are in parentheses).

Comparison of the two experiments suggests that it is more difficult to learn discordant relationships compared to concordant. To verify this, we contrasted performance on trials in which the second stimulus was matched between concordant and discordant conditions. For example, a concordant trial 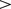 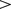 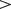 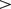 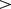 followed by 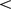 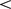 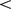 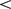 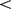 from Experiment 1 was matched with the discordant trial 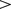 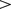 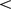 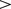 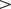 followed by 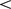 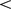 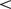 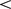 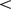 from Experiment 2; performance on the second stimuli was then compared. As before, only trials 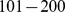 were used, and outliers were dropped (see Table 3). A two-way mixed-design ANOVA (between-subjects factor: concordant vs. discordant condition; within-subjects factor: congruent vs. incongruent trials) revealed a main effect of congruency [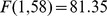, 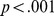] and of condition [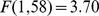, 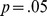] for accuracy. For RT, there was a main effect of congruency [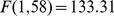, 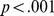] and of condition [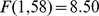, 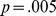]. Thus, across the four stimulus types there was a significant difference in accuracy and in RT between concordant and discordant trials. Participants responded faster to the second stimulus of a pair when the congruence of the first stimulus reliably matched the congruence of the second; in contrast, the second stimulus received no such performance boost when the congruence of the first stimulus reliably *mis*matched the congruence of the second. There was also reliably greater accuracy for concordant trials in which the second stimulus was incongruent.

**Table 3:** Average accuracy (%) and RT (ms.) for the second stimulus in concordant and discordant pairs.

	Concordant		Discordant		Significance	
2nd stim.	Accu.	RT	Accu.	RT	Accu.	RT
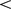 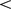 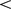 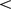 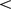	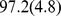	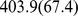	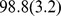	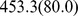	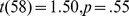	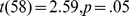
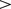 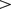 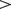 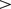 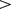	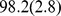	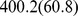	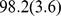	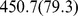	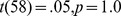	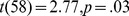
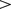 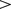 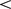 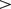 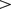	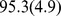	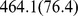	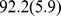	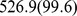	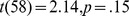	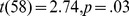
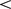 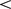 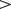 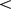 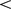	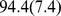	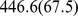	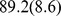	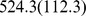	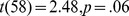	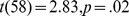

(Standard deviations are in parentheses; 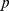 values are corrected for multiple-comparisons using the Bonferroni method).

Why did participants fail to show improved performance for discordant trials? Either they failed to learn the statistical relationship between the two stimuli in a pair, or they failed to exploit the relationship despite learning it. To clarify the matter, we performed a third experiment in which participants were explicitly told about the statistical relationships between the two stimuli in a pair. This allowed us to examine whether having learned the regularities in advance would lead to a reduction in RT of the second stimulus in a pair.

## Experiment 3

Experiment 3 was like Experiment 2 except that participants were explicitly informed that every pair was discordant, that is, congruence was (invariably) followed by incongruence and vice versa. In a pilot study, we used 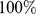 probability without explicit instructions, and the results were similar to those in Experiment 2. To ensure that participants have indeed learned the regularities, we used explicit instructions here. As before, half of the discordant trials involved a congruent stimulus followed by an incongruent stimulus, and the reverse for the other half. Since all trials were discordant, all trials were included in the analysis. Thirty new participants completed Experiment 3 (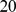 female, mean age 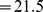 yrs, SD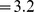).

### Results and Discussion

To determine whether participants exploited discordance in the present procedure, we compared performance on the second stimuli with performance on matching stimuli in the random condition of Experiment 1. As before, only trials 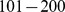 were included and RT outliers were excluded (only 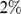 of the trials were removed as a result). The results are presented in Table 4.

**Table 4:** Average accuracy (%) and RT (ms.) for the second stimulus in discordant pairs in Experiment 3 compared to the random condition in Experiment 1.

Pair		Discordant		Random		Significance
1st stim.	2nd stim.	Accu.	RT	Accu.	RT	RT
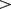 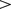 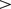 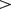 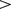	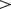 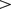 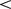 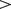 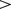	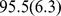	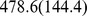	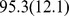	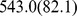	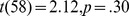
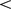 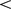 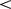 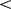 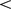	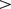 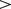 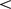 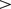 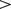	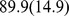	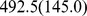	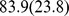	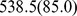	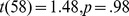
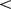 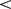 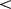 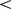 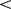	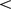 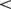 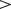 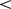 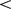	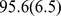	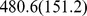	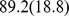	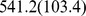	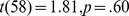
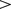 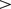 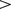 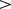 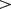	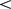 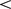 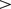 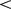 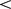	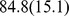	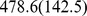	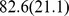	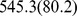	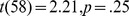
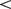 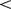 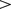 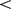 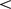	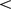 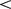 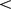 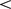 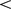	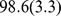	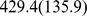	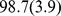	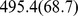	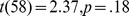
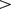 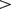 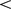 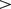 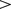	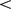 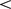 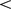 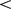 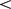	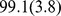	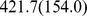	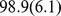	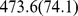	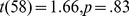
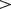 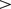 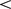 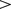 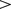	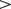 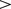 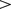 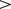 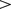	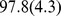	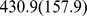	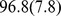	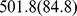	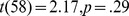
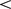 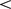 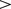 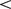 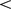	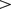 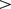 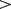 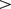 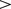	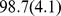	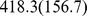	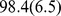	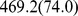	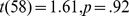

(Standard deviations are in parentheses; 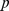 values are corrected for multiple-comparisons using the Bonferroni method).

As before, accuracy and RT were analyzed with a two-way mixed-design ANOVA (between-subjects factor: discordant vs. random condition; within-subjects factor: congruent vs. incongruent trials). For RT, there was a main effect of condition [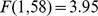, 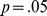], and also of congruency [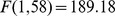, 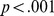]. For accuracy, there was a main effect of congruency [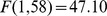, 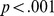], but not of condition [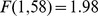, 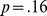]. There was no reliable interaction for accuracy or RT [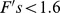, 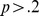]. We also compared the present results with those in Experiment 2, using a two-way ANOVA (between-subjects factor: discordant (explicit) vs. discordant (implicit) vs. random; within-subjects factor: congruent vs. incongruent). For accuracy, there was a main effect of congruency [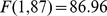, 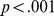], but not of condition [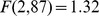, 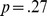]. For RT, there was a main effect of congruency [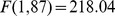, 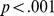], but a marginal main effect of condition [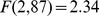, 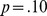]. There was no reliable interaction for accuracy or RT [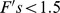, 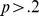]. This suggests that learning the discordant structure via explicit instructions resulted in faster RTs, compared to implicit learning of discordance.

It can be seen that RTs in the present experiment were reliably lower for most stimulus types compared to the random condition of Experiment 1. The enhanced performance seems not to be due to delaying the response to first stimuli. The average RTs of the first stimuli in the present condition and in the random condition of Experiment 1 were 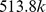 (SD = 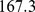) and 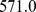 (SD = 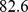), respectively, not reliably different [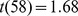, 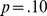]. We also note that there was no reliable difference in accuracy between the two conditions for any type of discordant trial. Thus, having been informed ahead of time about the regularities in discordant pairs resulted in faster RTs in the second stimulus. This suggests that the lack of improvement in RT in Experiment 2 is due to a failure to learn the statistical regularities of discordant pairs, rather than a failure to exploit the regularities despite learning them.

## General Discussion

Our first experiment documents the control over attention that observers can exercise when they learn that the congruency of one stimulus tends to match that of a successor. Importantly, this effect is not due to repetition priming since the response required by either stimulus in a concordant pair was randomly determined. The unanimous reduction in RT in the second stimulus across all types of trials suggests that the effect is driven by the learning of the concordant structure. That is, a congruent stimulus tends to be followed by another congruent stimulus, or an incongruent stimulus followed by another incongruent one. Remarkably, such learning occurred at an implicit level, because the vast majority of participants were unaware of the concordant structure. Presumably, the control is based on extending attention to the flankers for upcoming congruent stimuli and restricting it for incongruent. The same RT effect was observed for discordant trials, where the congruency of the first stimulus mismatches that of the second, but only when such structure was explicitly mentioned (Experiment 3). In contrast, discordance (mismatch of congruency) was difficult to learn implicitly (Experiment 2).

Our results suggest that cognitive control, a process which usually involves the active maintenance of explicit goals in working memory, can be governed by the implicit learning of stimulus relationships. Unlike most studies on cognitive control, participants in Experiment 1 were not informed about concordance, and instead learned the regularities spontaneously during the experiment. Such learning can be highly ecological and useful, suggesting that the existence of environmental regularities can allow participants to automatically increase their attentional control, even in the absence of explicit instructions or awareness.

The current results also suggest that the Gratton effect can be driven by a failure in learning the discordant structure, rather than a failure to exploit the regularities despite learning them. Regardless of concordance or discordance, RT declined for all types of trials when the congruency in the first stimulus predicted that of the second stimulus implicitly in Experiment 1 and explicitly in Experiment 3. This is consistent with recent findings on how the congruency of the previous stimuli influences performance on the upcoming trial, and how increasing the amount of concordant or discordant trials influences RT [9], [10].

It remains to understand why implicit learning is impeded by mismatching congruency between stimuli whereas it is possible when congruency matches (Experiment 2 versus 1). The two situations, after all, convey equivalent information. Several factors can potentially explain such failure in learning. First, it may be more difficult to perceive discordance than concordance [11], [12], the former involving a change in stimulus property (e.g., congruency predicts incongruence), whereas the latter involving no such change (e.g., congruency predicts congruency). Second, even if discordance can be perceived, it may be more effortful to switch between attending narrowly to the central arrow and attending broadly to the whole stimulus, as evidenced by various task switching costs in previous studies [8], [13]. This switch cost may not allow learning to be fully expressed. Third, since a discordant pair always involves the alternation between a congruent and an incongruent stimulus, such alternation could impede feature integration on the second stimulus [14] or result in negative priming [5], [6], which can prevent the learning of regularities between stimuli. Specifically, feature integration on discordant pairs may slow down performance because partial feature repetition in the second stimulus may require the feature binding in the first stimulus to be undone. This account could account for the differences in RT between concordant and discordant pairs (see Table 3). Finally, since cognitive control can be configured by the occurrence of conflict [15], attentional control may be increased following high-conflict incongruent trials, and yet decreased following low-conflict congruent trials. This suggests that incongruent trials can trigger a narrowing of attention which would in turn lead to faster RTs for upcoming incongruent trials, while congruent trials can induce a broadening of attention which would lead to faster RTs for upcoming congruent trials but slower RTs for incongruent trials. This may explain the faster RTs in concordant trials in Experiments 1 and slower RTs in discordant trials in Experiment 2. Further studies are needed to tease these ideas apart.

Other future research directions might profitably examine the generalizability of implicit learning of stimulus contingency on cognitive control [16], by using numeric stimuli or embedding new kinds of structure in the trial sequence. In the current study, the stimuli were grouped in pairs. This raises the question of how the history of congruency (e.g., the last five trials vs. the last ten trials) influences attentional control on upcoming trials. Finally, it remains to be seen how long the effects of implicit learning last on modulating cognitive control.
